# Efficacy and Safety of Alirocumab in Patients with Heterozygous Familial Hypercholesterolemia not Adequately Controlled with Current Lipid-Lowering Therapy: Design and Rationale of the ODYSSEY FH Studies

**DOI:** 10.1007/s10557-014-6523-z

**Published:** 2014-05-20

**Authors:** John J. P. Kastelein, Jennifer G. Robinson, Michel Farnier, Michel Krempf, Gisle Langslet, Christelle Lorenzato, Daniel A. Gipe, Marie T. Baccara-Dinet

**Affiliations:** 1 Department of Vascular Medicine, Academic Medical Center, University of Amsterdam, Meibergdreef 9, Room F4-159.2, 1105 AZ Amsterdam, The Netherlands; 2 University of Iowa, Iowa City, IA USA; 3 Point Medical, Dijon, France; 4 CHU de Nantes - Hopital Nord Laennec, Saint-Herblain, France; 5 Lipid Clinic, Oslo University Hospital, Oslo, Norway; 6 Sanofi, Paris, France; 7 Regeneron Pharmaceuticals, Inc., Tarrytown, NY USA; 8 Sanofi, Montpellier, France

**Keywords:** Alirocumab, Heterozygous familial hypercholesterolemia, LDL-C, PCSK9

## Abstract

**Background:**

Individuals with heterozygous familial hypercholesterolemia (heFH) have higher levels of low-density lipoprotein cholesterol (LDL-C) and are predisposed to premature cardiovascular disease. Alirocumab is a fully-human, monoclonal antibody targeted to proprotein convertase subtilisin/kexin type 9 currently in Phase 3 development for the treatment of hypercholesterolemia. Described here are three ODYSSEY Phase 3 trials, FH I (NCT01623115), FH II (NCT01709500) and HIGH FH (patients with heFH and LDL-C levels ≥160 mg/dL) (NCT01617655), in which alirocumab is further evaluated in the heFH population.

**Methods:**

Multicenter, multinational, randomized, double-blind, placebo-controlled studies have been designed to evaluate efficacy and safety of alirocumab in more than 800 patients with heFH who are not adequately controlled with a maximally-tolerated stable daily dose of statin for ≥4 weeks prior to the screening visit, with or without other lipid-lowering therapy. Patients are randomized (2:1) to receive alirocumab or placebo via a 1-mL subcutaneous auto-injection every 2 weeks (Q2W) for 78 weeks. In studies FH I and II, if their Week 8 LDL-C level is ≥70 mg/dL, patients will undergo a dose uptitration from 75 to 150 mg alirocumab Q2W at Week 12. In HIGH FH, patients will receive alirocumab 150 mg Q2W throughout the entire treatment period. The primary efficacy endpoint in all three studies is the percent change in calculated LDL-C from baseline to Week 24.

**Conclusions:**

The ODYSSEY FH studies are three Phase 3 studies aiming to further evaluate the efficacy and long-term safety of alirocumab as an effective therapeutic option for patients with heFH.

## Introduction

Heterozygous familial hypercholesterolemia (heFH) is a hereditary lipid metabolism disorder that predisposes affected individuals to cardiovascular (CV) disease [1]. Patients with heFH typically have very high low-density lipoprotein cholesterol (LDL-C) levels—often >190 mg/dL at the time of diagnosis—that are associated with high risk for premature CV disease [2, 3]. However, a recent consensus statement highlights that in most countries less than 1 % of patients with heFH are, in fact, diagnosed [4].

Findings from observational studies have shown that the risk of coronary heart disease (CHD) is reduced in heFH patients receiving statin therapy [5–7]; however, even with this treatment, the risk of CHD is still greater in heFH patients than in the general population [5]. Despite the availability of lipid-lowering therapy (LLT), approximately 80 % of patients with heFH do not reach the recommended levels of LDL-C [8–12]. Given the increased CV risk in the heFH population, there is a need to provide patients with additional and more intensive lipid-lowering therapy [4, 13].

Proprotein convertase subtilisin/kexin type 9 (PCSK9), a therapeutic target currently under investigation, binds to LDL-C receptors, resulting in their degradation so that fewer receptors are available on liver cells to remove excess LDL-C from the plasma [14–17].

PCSK9 inhibition has the potential to provide a complementary mechanism to other LLTs to significantly reduce LDL-C beyond the efficacy of statins [15, 17–25]. Alirocumab (formerly REGN727/SAR236553; Sanofi-Regeneron) is a fully human, monoclonal antibody targeted to PCSK9 currently in Phase 3 development for the treatment of hypercholesterolemia.

In Phase 2 trials, alirocumab demonstrated significant reductions in LDL-C levels in patients receiving concomitant statin or statin plus ezetimibe therapy [NCT01288443; NCT01266876; NCT01288469; 21, 23, 24]. In these studies, alirocumab significantly (*p* < 0.001) reduced mean LDL-C levels by up to 72.4 %, and also showed favorable trends in other atherogenic lipid parameters including lipoprotein(a) [Lp(a)], triglycerides, non-high-density lipoprotein cholesterol (non-HDL-C), apolipoprotein (Apo) B, and anti-atherogenic fractions HDL-C and Apo A1. Specifically, a Phase 2 trial demonstrated that alirocumab reduced mean LDL-C levels by up to 67.9 % (*p* < 0.0001) in patients with heFH [24]. No dose-limiting adverse events (AEs) were identified in the Phase 2 studies [21, 23, 24], with the most common treatment-emergent AE (TEAE) in patients receiving alirocumab being mild injection site reaction. Five serious AEs (SAEs) occurred in four patients (1.5 %) who received alirocumab and two SAEs occurred in two patients (2.6 %) who received placebo.

The ODYSSEY Phase 3 alirocumab clinical trial program is designed to further assess the efficacy and safety of alirocumab in a range of clinical settings. The trials within the program evaluate a treat-to-goal approach, using a flexible dosing strategy for individualized therapy based on degree of LDL-C lowering required to achieve an adequate treatment response. This approach is designed to address patient populations unable to achieve desired LDL-C levels with the current standard of care. The program comprises a total of 14 studies planned to include more than 23,500 patients in over 2,000 study centers worldwide. Of these studies, the ODYSSEY program also includes a large cardiovascular outcomes trial evaluating the long-term impact of alirocumab and lower levels of LDL-C on the occurrence of cardiovascular events in 18,000 patients after a recent (<52 weeks) acute coronary syndrome event, with a randomized treatment period of 64 months. The aim of the ODYSSEY FH studies is to assess the efficacy and safety of alirocumab in patients with heFH who, despite their maximally-tolerated statin dose, with or without other LLT, continue to have suboptimal LDL-C levels and require additional pharmacologic management.

## Methods

### Study Design

All three Phase 3 ODYSSEY FH clinical trials are multicenter, multinational, randomized, double-blind, placebo-controlled studies designed to evaluate the efficacy and safety of alirocumab in patients with heFH not adequately controlled with their current LLT.

The FH I study (ClinicalTrials.gov identifier: NCT01623115) is being conducted at 89 sites across North America, Europe and South Africa, and has a planned population of 471 patients. The FH II study (ClinicalTrials.gov identifier: NCT01709500) is being conducted at 26 sites across Europe and has a planned population of 250 patients.

**Fig. 1:**
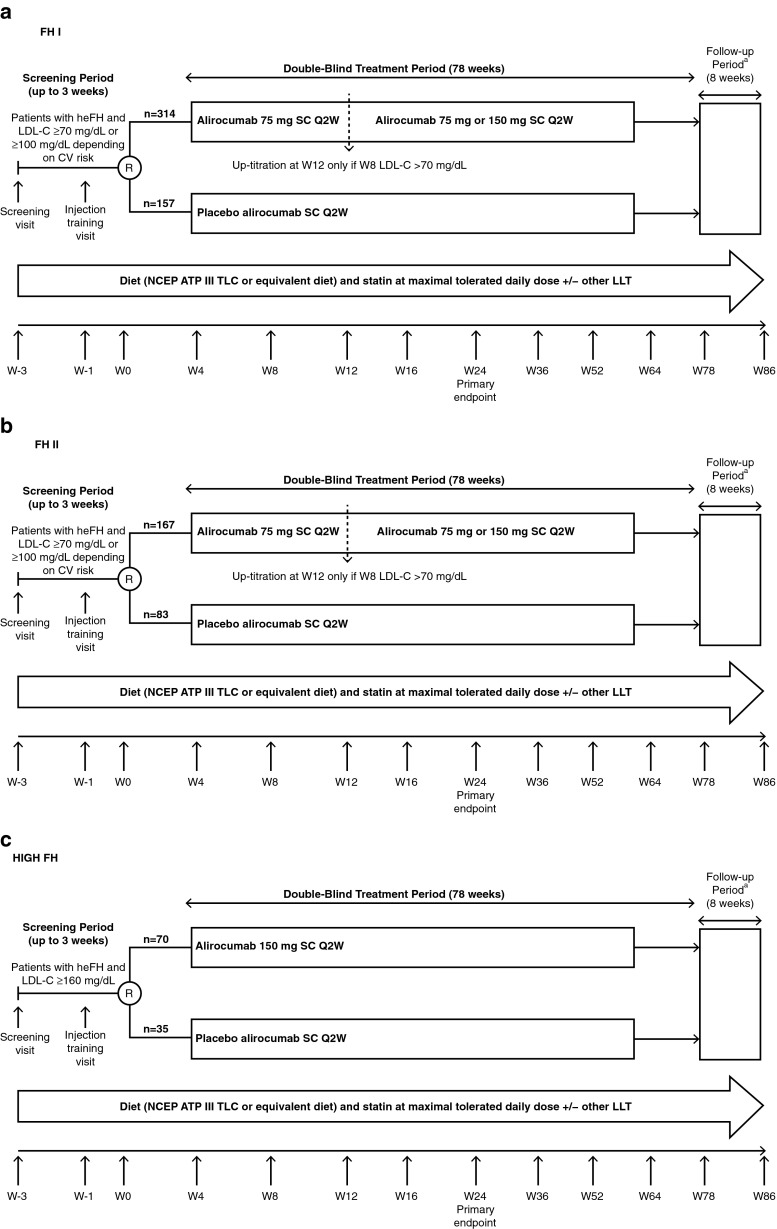
Study designs. **a** ODYSSEY FH I, **b** ODYSSEY FH II and **c** ODYSSEY HIGH FH. *CV* cardiovascular, *heFH* heterozygous familial hypercholesterolemia, *LDL-C* low-density lipoprotein cholesterol, *LLT* lipid-lowering therapy, *NCEP ATP III TLC* National Cholesterol Education Program Adult Treatment Panel III Therapeutic Lifestyle Changes, *Q2W* every 2 weeks, *R* randomization, *SC* subcutaneous. ^a^At the end of the double-blind treatment period, patients will be offered the possibility to enter an open-label extension study, in which they will receive alirocumab. If patients opt out of entering the open-label treatment period, they will enter the 8-week follow-up period

Both the FH I and II studies enroll heFH patients who have LDL-C levels ≥70 mg/dL at the screening visit and have a history of documented CVD, or heFH patients with LDL-C ≥100 mg/dL at the screening visit without a history of documented CVD (Fig. 1).

The HIGH FH study (ClinicalTrials.gov identifier: NCT01617655) is being conducted at 33 sites across North America, Europe and South Africa, and has a planned population of 105 patients with heFH and LDL-C levels ≥160 mg/dL at the screening visit (Fig. 1).

The studies are being performed in accordance with the ethical principles that have their origin in the Declaration of Helsinki and all applicable amendments laid down by the World Medical Assemblies and the International Conference Harmonisation guidelines for Good Clinical Practice. Institutional review board or independent ethics committee approval of the protocols and informed consent forms were obtained from each study site, and written informed consent was obtained from all patients.

### Study Objectives

The primary efficacy objective in all three studies is to demonstrate the reduction of LDL-C by alirocumab, in comparison with placebo, as add-on therapy to stable, maximally tolerated daily statin therapy, with or without other LLT, after 24 weeks of treatment in patients with heFH. Secondary objectives common to all three studies include the evaluation of the effect of alirocumab in comparison with placebo on LDL-C after 12 weeks of treatment; the effect of alirocumab on other lipid parameters, e.g., Apo B, non-HDL-C, total cholesterol, Lp(a), HDL-C, triglycerides and Apo A1 levels; the long-term effect of alirocumab on LDL-C; the safety and tolerability of alirocumab; and evaluation of the development of anti-alirocumab antibodies.

### Inclusion Criteria

The principal inclusion criteria for all three studies are patients with heFH who are not adequately controlled with a maximally tolerated stable daily dose of statin for at least 4 weeks prior to the screening visit, with or without other LLT (Table 1).

**Table 1:** Principal inclusion and exclusion criteria for ODYSSEY FH I, FH II, and HIGH FH

Principal inclusion criteria for all three studies	
Patients with heFH who are not adequately controlled^a^ with a maximally-tolerated stable daily dose of statin^b^ for at least 4 weeks prior to the screening visit, with or without other LLT	
Principal exclusion criteria	
Not on a stable dose of LLT (including statin) for at least 4 weeks and/or fenofibrate for at least 6 weeks, as applicable, prior to the screening visit or from screening to randomization	
Currently taking a statin that is not simvastatin, atorvastatin or rosuvastatin taken daily at a registered dose	
Receiving daily doses above atorvastatin 80 mg, rosuvastatin 40 mg or simvastatin 40 mg (except for patients on simvastatin 80 mg for more than 1 year, who are eligible)	
Use of fibrates, other than fenofibrate, within 6 weeks of the screening visit	
Fasting serum triglycerides >400 mg/dL (>4.52 mmol/L) at the screening visit	
Known history of homozygous FH	
FH I and II	HIGH FH
History of documented CVD and LDL-C <70 mg/dL (<1.81 mmol/L) at the screening visit Without history of documented CVD and LDL-C <100 mg/dL (<2.59 mmol/L) at the screening visit	LDL-C <160 mg/dL (<4.14 mmol/L) at the screening visit AND patient only on statin monotherapy without additional LLT

*CVD* cardiovascular disease, *FH* familial hypercholesterolemia, *heFH* heterozygous familial hypercholesterolemia, *LDL-C* low-density lipoprotein cholesterol, *LLT* lipid-lowering therapy, *MI* myocardial infarction

^a^FH I and II: not adequately controlled defined as an LDL-C ≥70 mg/dL at the screening visit in patients with a history of documented CVD OR LDL-C ≥100 mg/dL at the screening visit in patients without a history of documented CVD. HIGH FH: not adequately controlled defined as an LDL-C ≥160 mg/dL at the screening visit

^b^Maximally-tolerated statin dose defined as: the highest tolerable registered dose of daily statin currently administered to the patient, that is rosuvastatin 20 mg or 40 mg daily; atorvastatin 40 mg or 80 mg daily; simvastatin 80 mg daily (if already on this dose for >1 year). Patients who are not able to be on any of the above statin doses should be treated with the dose of daily atorvastatin, rosuvastatin, or simvastatin which is considered appropriate for the patient, according to the investigator’s judgment

The diagnosis of heFH must be made either by genotyping or clinical criteria. For those patients not genotyped, the clinical diagnosis may be based on either the Simon Broome criteria [26], meeting the criteria for definite FH, or the World Health Organization (WHO)/Dutch Lipid Network criteria [27] with a score of >8 points.

### Exclusion Criteria

The principal exclusion criteria for the FH I and II studies include patients with different LDL-C thresholds depending on their cardiovascular risk status: LDL-C <70 mg/dL (<1.81 mmol/L) in patients with history of documented CVD and LDL-C <100 mg/dL (<2.59 mmol/L) in patients without history of documented CVD.

The principal exclusion criterion for the HIGH FH study includes patients with an LDL-C level of <160 mg/dL (<4.14 mmol/L) at the screening visit (Table 1). Other key exclusion criteria common to the three studies are shown in Table 1.

### Study Procedures

The three FH studies consist of three periods: a screening period of up to 3 weeks, during which the patient or another designated person is trained to self-inject/inject study medication (1 mL administered via auto-injector); a 78-week double-blind treatment period; and an 8-week off-treatment follow-up period. It should be noted that, at the end of the 78-week treatment period in each study, patients will be offered the possibility to enter an open-label extension study, in which they will receive alirocumab. If patients opt out of entering the open-label treatment period, they will enter the 8-week follow-up period.

In FH I, FH II and HIGH FH, all eligible patients are randomized 2:1 to alirocumab or placebo. Randomization is stratified according to history of myocardial infarction (MI) or ischemic stroke for balance across study arms, intensity of statin treatment (atorvastatin 40 to 80 mg daily, or rosuvastatin 20 to 40 mg daily versus simvastatin irrespective of the daily dose, atorvastatin below 40 mg daily or rosuvastatin below 20 mg daily), and geographic region (where applicable).

At randomization, treatment kit numbers are allocated using a centralized treatment allocation system, which is either an interactive voice response system or an interactive web response system, depending on the site preference. Study patients, principal investigators and study site personnel remain blinded to all randomization assignments throughout the study. All lipid and anti-drug antibody results collected after randomization are masked.

In the three studies, the first auto-injection during the double-blind treatment period takes place at the site on the day of randomization (Week 0). Subsequent auto-injections are done by the patient or another designated person such as a spouse or relative at a patient-preferred location and occur every 2 weeks.

In FH I and FH II, patients randomized to alirocumab will receive a 75 mg 1 mL dose every 2 weeks (Q2W). Patients randomized to placebo will receive a 1 mL subcutaneous placebo injection in an identical auto-injector to maintain blinding. At Week 12, patients randomized to alirocumab undergo a dose uptitration to 150 mg Q2W, also given as a 1 mL auto-injection, if the Week 8 LDL­C level is ≥70 mg/dL (1.81 mmol/L). The continuation of the 75 mg dose or dose up-titration to the 150 mg dose will occur in an automated process without site or patient awareness.

In HIGH FH, at study entry, patients have an LDL-C level of ≥160 mg/dL (4.14 mmol/L) and they will be randomized to receive a 150-mg 1 mL dose of alirocumab or placebo-alirocumab, without the uptitration process, from randomization to the end of the 78-week double-blind treatment period.

During the three FH studies, all patients are asked to follow a stable, National Cholesterol Education Program Adult Treatment Panel III Therapeutic Lifestyle Changes diet or equivalent throughout the entire study duration from screening to completion.

### Key Study Endpoints and Assessments

The primary efficacy endpoint in all three studies is the percent change in calculated LDL-C from baseline to Week 24, using all LDL-C values regardless of adherence to treatment (intent-to-treat [ITT] approach). Key secondary endpoints are summarized in Table 2.

**Table 2:** Primary and key secondary endpoints common to FH I, FH II and HIGH FH

Primary endpoint
% change in calculated LDL-C from baseline to Week 24 in the ITT population, using all LDL-C values regardless of adherence to treatment (ITT estimand)
Key secondary endpoints
% change in calculated LDL-C from baseline to Week 24 in the mITT population, using all LDL-C values during the efficacy treatment period (on-treatment estimand)
% change in LDL-C from baseline to Week 12 (ITT estimand)
% change in LDL-C from baseline to Week 12 (on-treatment estimand)
% change in Apo B from baseline to Week 24 (ITT estimand)
% change in Apo B from baseline to Week 24 (on-treatment estimand)
% change in non-HDL-C from baseline to Week 24 (ITT estimand)
% change in non-HDL-C from baseline to Week 24 (on-treatment estimand)
% change in total cholesterol from baseline to Week 24 (ITT estimand)
% change in Apo B from baseline to Week 12 (ITT estimand)
% change in non-HDL-C from baseline to Week 12 (ITT estimand)
% change in total cholesterol from baseline to Week 12 (ITT estimand)
% change in calculated LDL-C from baseline to Week 52 (ITT estimand)
Proportion of very high CV risk patients reaching calculated LDL-C <70 mg/dL (1.81 mmol/L) or high CV risk patients reaching calculated LDL-C <100 mg/dL (2.59 mmol/L) at Week 24 (ITT estimand)
Proportion of very high CV risk patients reaching calculated LDL-C < 70 mg/dL (1.81 mmol/L) or high CV risk patients reaching calculated LDL-C <100 mg/dL (2.59 mmol/L) at Week 24 (on-treatment estimand)
Proportion of patients reaching LDL-C <70 mg/dl (1.81 mmol/L) at Week 24 (ITT estimand)^a^
Proportion of patients reaching LDL-C <70 mg/dl (1.81 mmol/L) at Week 24 (on-treatment estimand)^a^
% change in Lp(a) from baseline to Week 24 (ITT estimand)
% change in HDL-C from baseline to Week 24 (ITT estimand)
% change in fasting TG from baseline to Week 24 (ITT estimand)
% change in Apo A1 from baseline to Week 24 (ITT estimand)
% change in Lp(a) from baseline to Week 12 (ITT estimand)
% change in HDL-C from baseline to Week 12 (ITT estimand)
% change in fasting TG from baseline to Week 12 (ITT estimand)
% change in Apo A1 from baseline to Week 12 (ITT estimand)

*Apo* apolipoprotein, *FH* familial hypercholesterolemia, *HDL-C* high-density lipoprotein cholesterol, *ITT* intent-to-treat, *LDL-C* low-density lipoprotein cholesterol, *Lp(a)* lipoprotein(a), *mITT* modified ITT, *TG* triglycerides

^a^Proportion of patients reaching LDL-C <70 mg/dL (ITT and on-treatment estimand) are the last key secondary endpoints for HIGH FH

In FH I, II and HIGH FH, LDL-C will be calculated using the Friedewald formula at screening and at all time points during the double-blind treatment periods. In FH I and II, if triglycerides exceed 400 mg/dL (4.52 mmol/L) then the central laboratory will reflexively measure LDL-C (via the beta quantification method) rather than calculating it. LDL-C will also be measured (via the beta quantification method) at Week 0 and Week 24.

On-site patient assessments take place at Weeks 0, 12, 24, 36, 52, 64, 78, and 86, the end-of-study visit. All laboratory tests are performed by the central laboratory.

Safety parameters (AEs [including adjudicated CV events categorized as CHD death, non-fatal MI, fatal and non-fatal ischemic stroke, unstable angina requiring hospitalization, congestive heart failure requiring hospitalization], laboratory data [blood biochemistry, hematology and urinalysis], vital signs and electrocardiogram) are assessed throughout the study.

### Statistical Design and Analysis

#### Sample Size Determination

In each study (FH I, FH II and HIGH FH), a total sample size of 45 patients (30 in the alirocumab and 15 in the placebo group) will have 95 % power to detect a difference in mean percent change in LDL-C of 30 % with a 0.05 two-sided significance level, assuming a common standard deviation of 25 % and all these 45 patients having an evaluable primary endpoint.

However, to meet regulatory requirements across the program, sample sizes were increased to assess the safety of alirocumab appropriately. Therefore, the final total sample sizes were increased and rounded to 471 in FH I, 250 in FH II and 105 in HIGH FH.

#### Analysis Populations for Each of the FH Studies

##### Primary Analysis

The primary analysis population will be the ITT population and will comprise all randomized patients with at least one baseline calculated LDL-C value available and at least one calculated LDL-C value available between Weeks 4 and 24 (regardless of treatment adherence). The percent change from baseline in LDL-C at Week 24 will be analyzed using a mixed effect model with repeated measures (MMRM) approach. All available post-baseline data from Week 4 to 24 (on- and off-treatment) will be used, in which missing data will be accounted for by the MMRM [28, 29]. The model includes fixed categorical effects of treatment group, randomization strata time point, treatment-by-time point and strata-by-time point interaction as well as the continuous fixed covariates of baseline LDL-C value and baseline value-by-time point interaction.

##### Secondary Analysis

A hierarchical procedure will be used to control type I error and handle multiple secondary endpoints analyses. If the primary endpoint analysis (ITT) is significant at 5 % alpha level, key secondary efficacy endpoints will be tested sequentially in the order given in Table 2. In particular, LDL-C reduction at Week 24 will be analyzed on-treatment in the modified ITT population (i.e. patients with at least one baseline and at least one calculated LDL-C value available on-treatment between Week 4 and 24) if the primary analysis is significant in the ITT population.

Continuous secondary endpoints, except Lp(a) and triglycerides, will be analyzed using the same MMRM model as for the primary endpoint. Lp(a) and triglycerides (which have a non-Gaussian distribution), and the binary secondary endpoints (proportion of patients with LDL-C <70 mg/dL and <100 mg/dL) will be analyzed using a multiple imputation approach for handling of missing values followed by robust regression [30] (for Lp(a) and triglycerides) or logistic regression (for binary endpoints).

#### Safety Analysis

AEs (including adjudicated CV events), laboratory parameters, and vital signs will be reported descriptively, based on the safety population (all randomized patients who received at least one dose or partial dose of study treatment). The safety analysis will focus on the TEAE period defined as the time from the first double-blind dose to the last double-blind dose of the investigational product +70 days (10 weeks).

Patients entering the open-label extension study will be followed up to their last visit in the double-blind treatment period and their TEAE period will be truncated at this visit.

##### Timing of Analysis

The analysis will be conducted in two steps. The first step will be the main efficacy and safety analyses conducted as soon as all patients have been randomized and have at least all their data up to Week 52 collected and validated. In this step, the final analysis of the primary and secondary efficacy endpoints up to Week 52 will be performed as well as the safety analysis on all safety data collected and validated at the time of the first analysis.

Of note, the results of the first analysis will not be used to change the conduct of the ongoing study in any aspect. Since the analyses of primary and key secondary efficacy endpoints will be final at the time of first step analysis, the significance level for the study remains at 0.05. The second step in the analysis will be conducted at the end of the study and will consist of the final analysis of Week 78 efficacy endpoints and the final safety analysis.

## Discussion

A recent consensus statement has been issued urging a worldwide need to address the underdiagnosis and undertreatment of patients with heFH [4]. The prevalence of heFH comes from estimates based on a theoretical frequency of 1/500 in the general population, and recent numbers indicate that approximately 689,900 individuals in the USA and Canada, 527,500 in Europe and 100,000 in South Africa have heFH [4]. Furthermore, some studies have indicated the prevalence can range from 1/500 to 1/200, and based on extrapolations, it is estimated that there are between 14 and 34 million individuals with heFH worldwide [4]. In many countries, however, less than 1 % of patients are diagnosed [4].

A diagnosis of heFH is typically made based on family history, clinical history of CHD, physical examination for xanthomas and corneal arcus, and very high levels of LDL-C; it can also be made by genotyping [31]. However, despite the increased understanding of genetic causes and direct detection of mutations in the LDLR, APOB, PCSK9 and LDLRAP genes, the vast majority of diagnoses still rely on the more historical clinical criteria.

Although it has been demonstrated that statin therapy, with or without other LLT, can reduce the risk of CHD in patients with heFH [5–7], many patients with heFH still do not achieve desired LDL-C levels [8–12, 22, 24, 32, 33] and more intensive treatment options for these high-risk patients are required [4]. To help address this unmet need, new approaches to lowering LDL-C, including monoclonal antibodies to PCSK9 administered as subcutaneous injection, are currently in development.

In evaluating the efficacy and safety of such antibodies to PCSK9, a Phase 2, randomized controlled trial of alirocumab conducted in patients with heFH and LDL-C levels >100 mg/dL despite their current LLT of a statin, with or without ezetimibe, showed that alirocumab was well tolerated and also showed statistically significant reductions in LDL-C levels at all doses of alirocumab compared with placebo [24]. With the large reductions in LDL-C, up to 94 % of patients achieved LDL-C levels <100 mg/dL and up to 81 % achieved levels <70 mg/dL [24]. In another Phase 2, randomized controlled trial with evolocumab in heFH patients with LDL-C levels >100 mg/dL despite receiving statin therapy, with or without ezetimibe, findings showed substantial reductions in LDL-C levels with evolocumab compared with placebo [22].

The ODYSSEY FH studies are Phase 3 studies aiming to further demonstrate the potential of alirocumab as an effective therapeutic option for patients with this severe disorder. The uptitration dosing strategy in FH I and FH II allows for flexibility and individualized therapy based on the degree of LDL-C reduction required to achieve the desired treatment response. In HIGH FH, where patients entered the study with LDL-C values ≥160 mg/dL despite receiving a maximally tolerated statin dose, with or without ezetimibe, it was deemed appropriate to initiate the higher, 150 mg Q2W, dose of alirocumab. In addition, the option for patients to enter an open-label extension study at the end of the double-blind treatment period will allow for further assessment of the efficacy and safety of alirocumab.
